# Indigo carmine extravasation from intravenous injection caused by a blood pressure cuff

**DOI:** 10.1186/s40981-025-00835-3

**Published:** 2026-01-03

**Authors:** Masaya Sekiguchi, Jun Honda, Atsuyuki Hosono, Satoki Inoue

**Affiliations:** Department of Anesthesiology, Fukushima Medical University Hospital, 1 Hikarigaoka, Fukushima, Fukushima 960-1295 Japan

**Keywords:** Indigo carmine, Extravasation, Temporary blood flow occlusion, Cuff

To the Editor,

Intravenous administration of indigo carmine is commonly used to confirm the presence or absence of ureteral obstruction or injury. We here report a case of indigo carmine extravasation distal to the tourniquet site, which resulted from administration into a forearm vein during temporary blood flow occlusion by a proximal cuff applied on the same side.

A 69-year-old woman (157 cm, 36 kg) underwent an abdominal total hysterectomy with bilateral salpingo-oophorectomy for an ovarian tumor under general anesthesia. A blood pressure cuff was applied to her right upper arm, and blood pressure was measured at 5-minute intervals during the procedure. Two IV catheters were inserted, one into each forearm, and a check valve was attached to the 18-G IV catheter in the right forearm. Remifentanil and phenylephrine were administered continuously via the left forearm catheter, while other medications were administered intermittently via the right forearm catheter. During surgery, ephedrine, phenylephrine, and rocuronium administered intermittently via the peripheral venous line in the right arm were smoothly infused and produced their intended effects. After removal of the uterus and adnexa, 20 mg/5 ml of indigo carmine was administered via the right forearm catheter to check for ureteral injury, followed by 10 ml of normal saline. Blood pressure measured 1 min after indigo carmine administration was 84/63 mmHg. The patient’s urine was stained blue, with no dye leakage into the surgical field observed, and the procedure was concluded. After removing the dressing, pigment deposition was observed along the course of blood vessels distally from the cuff attachment site on the right forearm, extending even further distally than the intravenous insertion site (Fig. 1). Leakage of dye was observed not only in the vein where the intravenous line was placed, but also along the vein into the downstream veins and tributaries. There was no petechiae and evidence of extravasation at the right forearm venous access site. The patient recovered from anesthesia uneventfully, and the pigment deposition resolved within approximately 1 day.

**Fig. 1:**
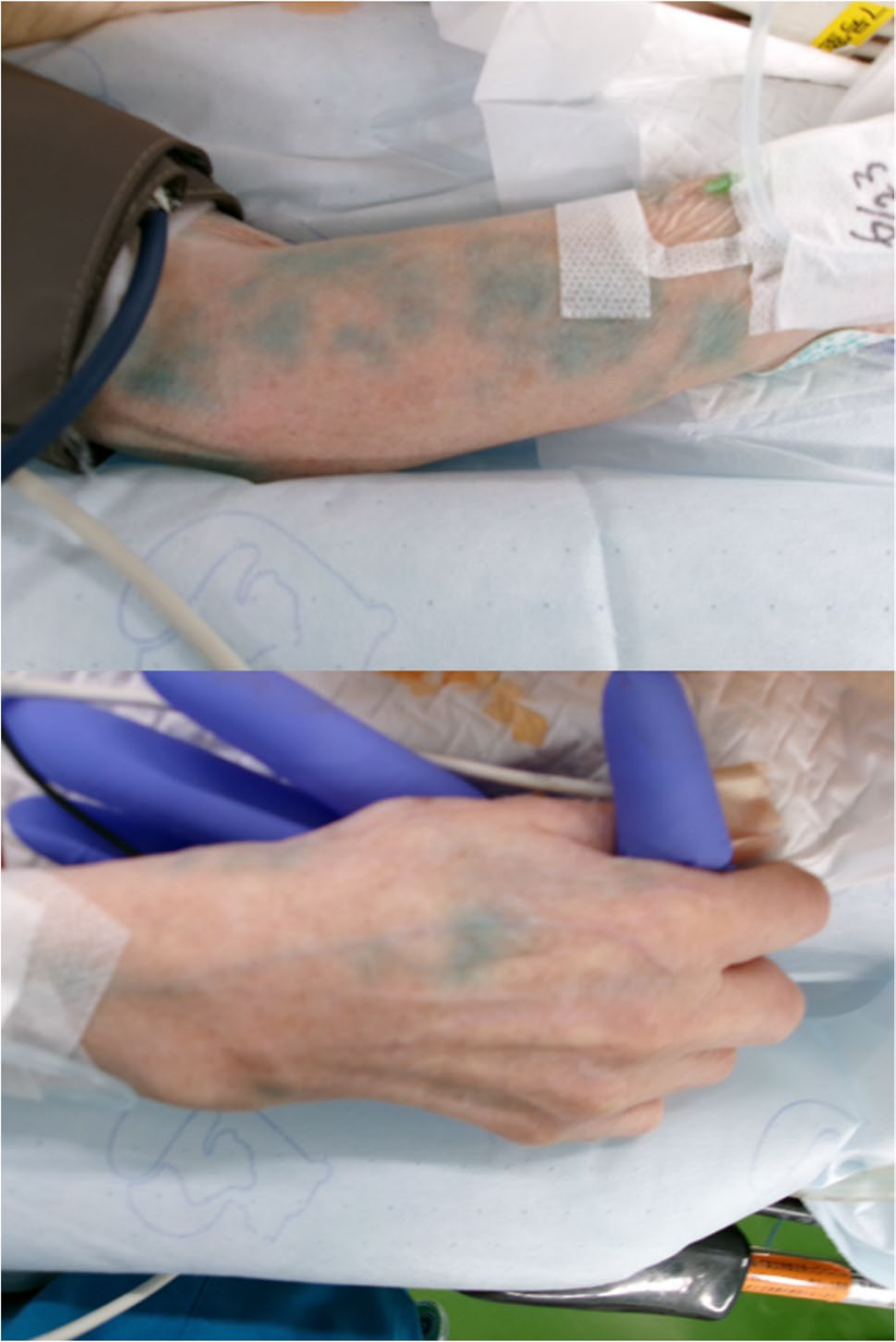
Indigo carmine leakage occurs along the course of vessels beyond the cuff and also along vessels beyond the venous line insertion site

To date, only one report has described indigo carmine leakage distal to a proximally applied cuff, despite correct IV catheter placement within the vessel [1]. The Bier block, also known as intravenous regional anesthesia, is a technique where a tourniquet is applied proximal to a venous line placed in a limb [2], thereby confining the injected local anesthetic within the vasculature to achieve anesthesia of the limb. In the present case, extravasation occurred despite both the tourniquet time and drug dose being lower than those typically used in a Bier block procedure.

Although it is common to insert intravenous catheters into both upper limbs or to place a venous line on the same side as the cuff, reports of extravasation as seen in the present case are rare. Vascular permeability is known to increase with blood stasis, inflammation, tumors, or wound healing [3, 4], and it cannot be ruled out that the patient’s predisposing factors influenced this outcome. While no adverse events have been reported in cases of indigo carmine extravasation, such events may occur with other agents. Unlike indigo carmine, which is a dye and thus visible upon extravasation, other agents may go unnoticed, potentially leading to delayed detection and subsequent complications. Anesthesiologists should be aware that even brief blood pressure measurements using a cuff pose a risk of extravasation of intravenously administered agents.

## Data Availability

Not applicable.
